# TNFα-Mediated Priming of Mesenchymal Stem Cells Enhances Their Neuroprotective Effect on Retinal Ganglion Cells

**DOI:** 10.1167/iovs.61.2.6

**Published:** 2020-02-07

**Authors:** Ben Mead, Xitiz Chamling, Donald J. Zack, Zubair Ahmed, Stanislav Tomarev

**Affiliations:** 1 School of Optometry and Vision Sciences, Cardiff University, Cardiff, United Kingdom; 2 Neuroscience and Ophthalmology, Institute of Inflammation and Ageing, University of Birmingham, Birmingham, United Kingdom; 3 Section of Retinal Ganglion Cell Biology, Laboratory of Retinal Cell and Molecular Biology, National Eye Institute, National Institutes of Health, Bethesda, Maryland, United States; 4 Wilmer Eye Institute, Departments of Ophthalmology, Molecular Biology and Genetics, Neuroscience, and Institute of Genetic Medicine, Johns Hopkins University School of Medicine, Baltimore, Maryland, United States

**Keywords:** exosomes, extracellular vesicles, TNFα, mesenchymal stem cells, glaucoma, priming, retinal ganglion cell

## Abstract

**Purpose:**

To determine whether priming of bone marrow mesenchymal stem cells (MSCs) by signals from injured retina, particularly tumor necrosis factor α (TNFα), increase their exosomes’ neuroprotective efficacy on retinal ganglion cells (RGCs).

**Methods:**

MSCs were primed with retinal cell culture conditioned medium, with or without the TNFα blocker etanercept or TNFα prior to isolation of exosomes. MSC conditioned medium or exosomes were added to rat retinal cultures or human stem cell–derived retinal ganglion cell (hRGC) cultures, and RGC neuroprotective effects were quantified. Luminex assays were used to compare primed versus unprimed exosomes.

**Results:**

MSC conditioned medium and exosomes exerted a significant neuroprotective effect on injured rat and hRGC. This effect was significantly increased after MSCs were primed with retinal conditioned medium or TNFα. Blocking of TNFα signaling with etanercept prevented priming-induced RGC neuroprotective efficacy. Priming increased Pigment epithelium-derived factor (PEDF) and VEGF-AA exosomal abundance.

**Conclusions:**

MSC exosomes promote RGC survival not just in rodent retinal cultures but also with hRGC. Their efficacy can be further enhanced through TNFα priming with the mechanism of action potentially mediated, at least in part, through increased levels of PEDF and VEGF-AA.

The retina is integral to transforming light information to an electrochemical signal and propagating this signal to the brain. Retinal ganglion cells (RGCs) are the final stage in the phototransductive pathway of the retina, and their axons make up the optic nerve. RGCs are acutely susceptible to optic nerve injury, and their loss, triggered by trauma or the degenerative neuropathies such as glaucoma, is one of the leading causes of vision loss worldwide.^1^ No neuroprotective treatments are currently available to prevent RGC degeneration and cell loss.

Bone marrow mesenchymal stem cells (MSCs), a self-renewing multipotent stem cell population, have demonstrated significant therapeutic efficacy in models of retinal trauma and disease.^2^ Intravitreal injection of MSCs yields a significant neuroprotective and axogenic effect in animal models of glaucoma ^3^^,^^4^ and other forms of optic neuropathy.^5^^–^^7^ Evidence strongly suggests that the mechanism of action is paracrine mediated, dependent on the expansive secretome of MSCs, which consists of multiple neurotrophic factors (NTFs) and exosomes.

While there is strong evidence that MSCs exert a significant effect on the retina through their signaling cytokines,^8^^,^^9^ signaling in the opposite direction (i.e., signaling from the injured retina onto MSCs) has not been well studied and remains poorly understood. It has been suggested that the expansive secretome of MSCs can be modulated by signaling molecules from the injured environment, activating or “priming” the MSCs and further upregulating their therapeutic potential. In retinal ischemia, where MSC conditioned medium provides a neuroprotective effect and can partially restore visual function when injected into the vitreous,^10^ the effect was significantly greater when MSCs had been subjected to hypoxic conditions prior to conditioning of culture,^11^ suggesting that they can enter a primed state in response to injury. Similar observations have been seen when treating an animal model of stroke with hypoxia-preconditioned MSCs.^12^

It is suggested that the inflammatory milieu is responsible for priming MSCs, and one candidate is tumor necrosis factor α (TNFα), a proinflammatory cytokine that is expressed by retinal glia and whose receptor, TNFα receptor 1, is expressed by RGCs.^13^ Retinal glial cells, when exposed to stressors simulating ischemia or ocular hypertension/glaucoma, upregulated and released TNFα.^14^^–^^17^ This increased TNFα expression is at least partially responsible for RGC death, and administration of TNFα neutralizing antibodies reduced RGC apoptosis.^18^ In rat^16^^,^^19^ and mouse^20^ models of ocular hypertension, significant elevations in retinal and optic nerve levels of TNFα were observed. In human patients with glaucoma, TNFα and TNFα receptor 1 were upregulated compared with age-matched normal donors.^17^^,^^21^^,^^22^ Recently, chronic ocular hypertension was shown to induce Müller cells to upregulate mRNA and protein expression of TNFα via ephrinB/EphB and downstream PI3K/Akt signaling.^14^

Our most recent work has demonstrated that MSC-derived exosomes exert a significant neuroprotective effect on RGCs.^23^^,^^24^ Exosomes are small extracellular vesicles that contain microRNA, mRNA, proteins, and lipids. There is still contention in the field on the appropriate terminology, whether they should be called exosomes or extracellular vesicles, with *exosomes* reserved for those preparations that have used extensive isolation and characterization steps.^25^ We use the term *exosome* throughout this article. Recent studies have shown that their internal cargo, both mRNA and proteins, can be modulated by injury.^26^ Exposure of endothelial cells to stressors such as hypoxia and inflammation led to significant changes in protein and mRNA abundance within subsequently isolated exosomes. The present study aimed to better understand the changes that MSC-derived exosomes undergo when exposed to the injured retinal environment and whether their therapeutic efficacy can be increased. We primed MSCs by pretreatment with dissociated retinal cell culture conditioned medium or TNFα. Primary rat retinal cultures and human stem cell–derived retinal cultures were then treated with MSC conditioned medium or MSC exosomes. MSC-exosomal proteins were analyzed, comparing unprimed MSCs to primed MSCs.

## Materials and Methods

**Figure 1.**
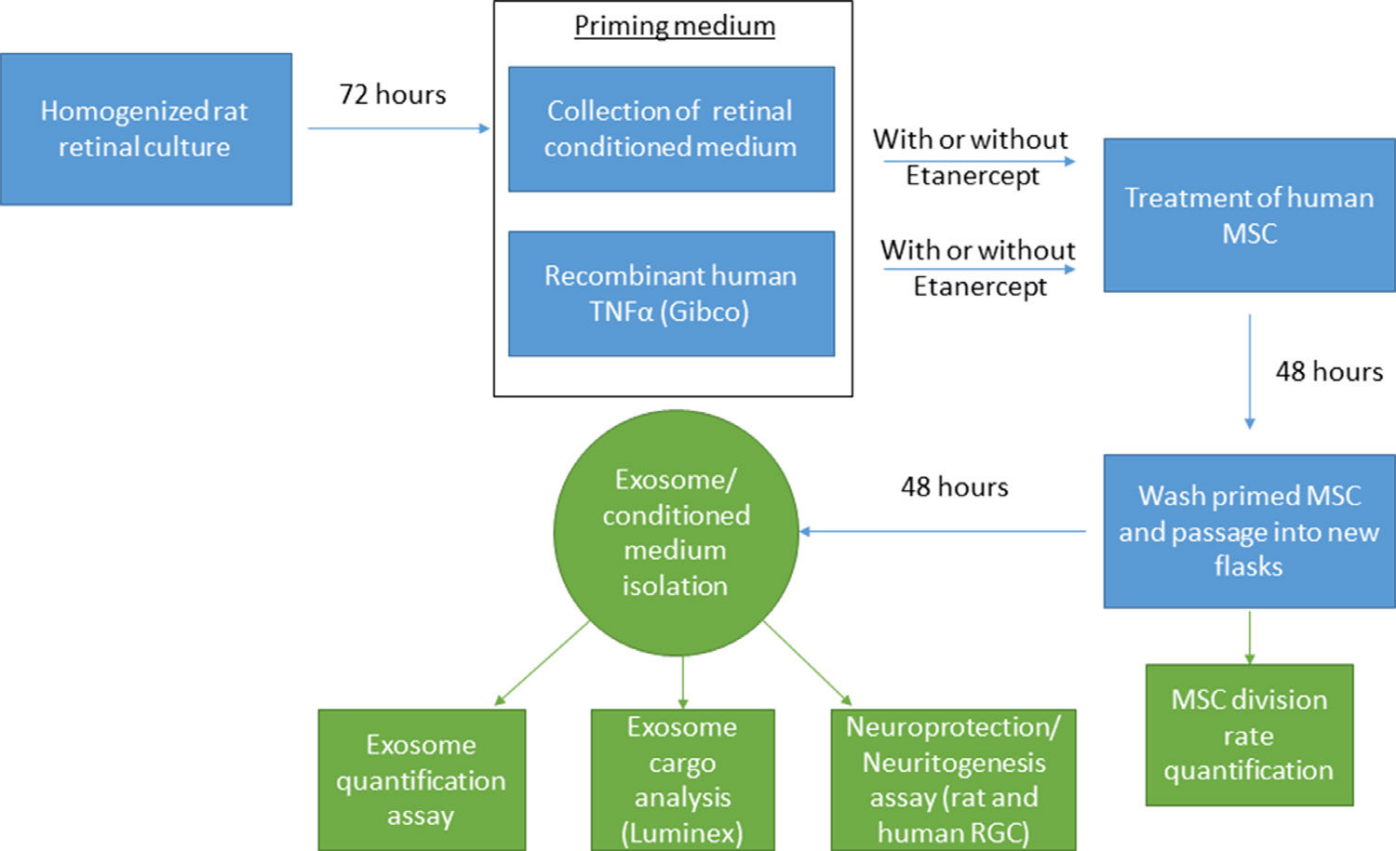
Overview of the Experimental Design.

All reagents were purchased from Sigma (Allentown, PA, USA) unless otherwise specified. See Figure 1 for an overview of the experimental design.

### MSC Cultures

Human MSCs (bone marrow derived) were purchased from Lonza (Walkersville, MD, USA) and represented pooled samples from three donors. CD29^+^/CD44^+^/CD73^+^/CD90^+^/CD45^−^ (confirmed by supplier) MSCs were seeded into T25 or T75 flasks, in a total volume of 5 mL or 15 mL Dulbecco's modified Eagle's medium (DMEM), respectively, containing 1% penicillin/streptomycin and 10% fetal bovine serum (Hyclone Laboratories, Logan, UT, USA) and at a quantity of 1 × 10^6^ cells and 2 × 10^6^ cells, respectively. Cultures were maintained at 37°C in 5% CO_2_, the supplemented medium was changed every three days, and the cells were passaged when 80% confluent using 0.05% trypsin/EDTA. For all experiments, MSCs were used at passages 2 to 5.

### Animals

Adult (10 weeks) female Sprague-Dawley rats weighing 170 to 200 g (Charles River, Wilmington, MA, USA) were housed under accordance with guidelines described in the ARVO Statement for the Use of Animals in Ophthalmic and Vision Research, using protocols approved by the National Eye Institute Committee on the Use and Care of Animals. These guidelines include conditions of 21°C and 55% humidity under a 12-hour light and dark cycle, food/water given ad libitum, and being under constant supervision from trained staff. Animals were killed by rising concentrations of CO_2_ before dissection of retinae.

### Retinal Cell Culture

Eight-well chamber slides (Thermo Fisher Scientific, Cincinnati, OH, USA) were precoated with 100 µg/mL poly-D-lysine for 60 minutes and then with 20 µg/mL laminin for 30 minutes. After culling and ocular dissection, the retinae of female Sprague-Dawley rats were minced in 1.25 mL papain (20 U/mL; Worthington Biochem, Lakewood, NJ, USA; as per manufacturer's instructions) containing 50 µg/mL DNase I (62.5 µL; Worthington Biochem) and incubated for 90 min at 37°C. The retinal cell suspension was centrifuged at 300 × *g* for 5 minutes and the pellet resuspended in 1.575 mL Earle's balanced salt solution (Worthington Biochem) containing 1.1 mg/mL reconstituted albumin ovomucoid inhibitor (150 µL; Worthington Biochem) and 56 µg/mL DNase I (75 µL). After adding to the top of 2.5 mL albumin ovomucoid inhibitor (10 mg/mL) to form a discontinuous density gradient, the retinal cell suspension was centrifuged at 70 × *g* for 6 minutes and the cell pellet resuspended in 1 mL of supplemented Neurobasal-A (25 mL Neurobasal-A; Life Technologies, Carlsbad, CA, USA), 1× concentration of B27 supplement (Life Technologies), 0.5 mM L-glutamine (62.5 µL; Thermo Fisher Scientific), and 50 µg/mL gentamycin (125 µL; Thermo Fisher Scientific). Retinal cells were then seeded into eight-well chamber slides at a concentration of 125,000 cells/well. After 72 hours in culture, retinal cell culture conditioned medium (from retinae collected from three animals) was collected to be used in MSC priming as below.

### MSC Priming

MSC cultures were primed by treating with retinal cell culture conditioned medium, prepared as above. Our previous studies have demonstrated that coculture of 50,000 MSCs with 125,000 dissociated retinal cells in 500 µL leads to significant neuroprotection and neuritogenesis.^7^^,^^8^ Therefore, 500,000 MSCs in a T75 flask were primed by treatment with conditioned medium from 1,250,000 dissociated retinal cells. The retinal cell culture conditioned medium was concentrated using Amicon 10,000 Molecular weight filters (Millipore, Burlington, MA, USA) prior to treatment of MSCs. This approach concentrated the medium by approximately 10-fold, meaning instead of adding 4 mL of retinal conditioned medium (from 1,250,000 dissociated retinal cells) to a single T75 flask containing MSCs, we only needed to add 400 µL.

In place of retinal cell culture conditioned medium, MSCs were also primed with recombinant human TNFα (Gibco, Cincinnati, OH, USA; 10ng/mL) for 48 hours.^27^ Retinal cell culture conditioned medium was also combined with the TNFα blocker, etanercept, at a concentration of 0.5 µg/mL according to previous dose-response studies in vitro.^28^

After culture in the priming medium (retinal conditioned medium or TNFα) for 48 hours, MSCs were washed in PBS three times, passaged, and grown in fresh DMEM containing 10% exosome depleted serum. After a further 48 hours in culture, MSC division rate was calculated and conditioned medium was collected (from three separate cultures) for the Luminex assay, exosome isolation, and assessment of neuroprotective/neuritogenic efficacy in retinal cultures.

### Exosome Isolation

Exosomes were isolated from MSCs using ExoQuick-TC (System Biosciences, Mountain View, CA, USA) per the manufacturer's instructions. Briefly, conditioned medium was centrifuged at 3000 × *g* for 15 minutes before incubation with ExoQuick reagent overnight at 4°C (1:10 ratio with medium). The solution was centrifuged at 1500 × *g* for 15 minutes a final time before resuspension of exosome pellet in sterile PBS. The exosome preparation was then passed through a 0.22-µm filter to remove large microvesicles and apoptotic bodies. Exosomes were characterized via Western blot by their positive staining for the exosome markers Syntenin 1 and CD63 and negative staining for high/low-density lipoprotein markers ApoA1 and ApoB.^24^ Briefly, exosomes were lysed in passive lysis buffer (Promega, Madison, WI, USA; #E1531) and protein samples (20 µg) were separated on 4% to 12% Bis-Tris protein gels at 150 V for 40 minutes. After transfer to polyvinylidene fluoride membranes and blocking in 10% Western blot blocking buffer (Roche, Basel, Switzerland) in Tris-buffered saline (TBS), proteins were stained overnight in primary antibody (Table) diluted in TBS. Samples were washed 3 × 5 minutes in Tris-buffered saline Tween (TBST), stained for 1 hour with secondary antibody (Table) in TBS, and washed 3 × 5 minutes in TBST before detection with Immobilon ECL reagents (Millipore, Burlington, MA, USA). Densitometry of Western blot bands was analyzed using ImageJ software (National Institutes of Health, Bethesda, MD, USA) as previously reported.^24^ All exosomes samples used in the study comprised exosomes pooled from three MSC donors.

**Table.** Antibodies Used in Immunocytochemistry (ICC) and Western Blot (WB)

Antigen	Dilution	Supplier	Catalogue No.
βIII-Tubulin	1:500 (ICC)	Sigma	#T-8660
Syntentin 1	1:1000 (WB)	Abcam	#Ab133267
CD63	1:1000 (WB)	System Bio	#Exoab-CD63-A1
ApoA1	1:1000 (WB)	Abcam	#ab7613
ApoB	1:1000 (WB)	Abcam	#ab20737
HSC70	1:5000 (WB)	Santa Cruz	#sc-7298
Mouse IgG HRP	1:2000 (WB)	GE Healthcare	#NA-931
Mouse IgG 488	1:400 (ICC)	Thermo Fisher	#A-11001
Mouse IgG HRP	1:2000 (WB)	GE Healthcare	#NA-931
Rabbit IgG HRP	1:10,000 (WB)	Cell Signalling	#7074

Horseradish peroxidase (HRP), XXX.

The concentration and size distribution of exosomes were characterized using a NanoSight LM10 instrument (Malvern, Worcester, MA, USA), equipped with a 405-nm LM12 module and EM-CCD camera (DL-658-OEM-630; Andor, Concord, MA, USA). Three videos were captured per sample with a camera level of 10. Videos were analyzed with a detection threshold of 2, automatic blur size, and 12.9–13.1 pix maximum jump size. Slider gain was set to 80 and a total of 567 frames were taken.

### Retinal Culture Treatment

To assess neuroprotective and neuritogenic effects of MSCs on RGCs, retinal cells were isolated as above and cultured at a density of 125,000 cells/300 µL/well in eight-well chamber slides. Retinal cultures were treated with MSC conditioned medium (primed or unprimed) and incubated for 72 hours at 37°C before fixation and immunocytochemical staining of RGCs with βIII-tubulin as detailed below. MSC conditioned medium treatments were 50 µL (concentrated using Amicon 10,000 Molecular weight filters) per well and derived from 50,000 MSCs over 48 hours. Selected cultures were treated with human recombinant Ciliary neurotrophic factor (CNTF) as a positive control (50 ng/mL). For this study, large spherical βIII-tubulin^+^ rat retinal cells,^29^ which can be identified by preferential βIII-tubulin staining intensity around the axonal base, are referred to as RGCs. Previous immunocytochemical analysis of these cultures in our laboratory demonstrates that 60% of these retinal cells are neurons (neurofilament^+^/βIII-tubulin^+^), of which 10% are Thy1^+^ RGCs and possess the unique βIII-tubulin staining pattern described above.^30^ Retinal cultures consisted of cultures from three different animals, three wells per animal.

### Human Stem Cell–Derived RGCs (hRGCs)

An H9-derived human embryonic stem cell line engineered such that tdTomato and mouse THY1.2 were knocked into the *POU4F2* (*BRN3B*) locus was differentiated into human RGCs (hRGCs) according to the published protocol.^31^^,^^32^ Briefly, cells are plated and grown on Matrigel (Corning, NY, USA) coated plates in mTeSR medium (Stem Cell Technologies, Vancouver, Canada) supplemented with blebbistatin (5 µM). Medium is switched on day 1 to retinal differentiation medium (RDM; DMEM/F12 + Neurobasal A medium [mixed 50:50], 1× Glutamax, 1× anti-anti, 1× N2 supplement, and 1× B27 supplement; Thermo Fisher Scientific) and cultured in this medium up until day 40. Small molecules are added as follows: days 1 to 6, dorsomorphin (1 µM), inducer of definitive endoderm 2 (2.5 µM; Stem Cell Technologies), forskolin (25 µM), and nicotinamide (10 µM); days 6 to 10, forskolin and nicotinamide; days 10 to 18, forskolin; days 18 to 30, forskolin and N-[N-(3,5-Difluorophenacetyl)-L-alanyl]-S-phenylglycine t-butyl ester (DAPT) (10 µM); and days 30 to 40, RDM alone. Note that the cultures contain hRGCs as well as other retinal cells but are called hRGCs for the sake of brevity.

Unlike adult rodent-derived RGC cultures that degenerate in culture without intervention, hRGCs do not degenerate unless triggered to do so. To induce stress, damage, and subsequent death, hRGCs were treated with 1 µM colchicine as previously described.^32^ Exosome treatment was given at the same time, and after 48 hours, cells were fixed as detailed below without the need for immunocytochemical staining. Three wells were used per treatment group and were repeated three times on separate days and with separate cultures.

### Immunocytochemistry

Retinal cells were fixed in 4% paraformaldehyde in PBS for 10 minutes, washed for 3 × 10 minutes in PBS, blocked in blocking solution (3% bovine serum albumin [g/mL], 0.1% Triton X-100 in PBS) for 20 minutes, and incubated with primary antibody (Table) diluted at 1:500 in antibody diluting buffer (ADB; 0.5% bovine serum albumin, 0.3% Tween-20 in PBS) for 1 hour at room temperature. Cells were then washed for 3 × 10 minutes in PBS, incubated with the secondary antibody (Table) diluted in ADB for 1 hour at room temperature, washed for 3 × 10 minutes in PBS, mounted in Vectorshield mounting medium containing DAPI (Vector Laboratories, Peterborough, UK), and stored at 4°C.

### Enzyme-Linked Immunosorbent Assay (ELISA)

To quantify the amount of TNFα released by cultured retina, conditioned medium from three separate cultures/animals was collected and assayed using a duoset ELISA kit according to the manufacturer's instructions (R&D Systems, Minneapolis, MN, USA). Briefly, a standard curve was constructed using TNFα standards and test samples of conditioned medium at increasing dilutions, run in duplicate with TNFα concentrations extrapolated from the standard curve.

### Multiplex Bead Immunoassay (Luminex)

Conditioned media from three separate cultures of MSCs and retina were analyzed using a neurotrophic multiplex Luminex kit (Thermo Fisher Scientific) according to the manufacturer's instructions and using the same principles described in the ELISA section. Assays were run using a Luminex-100 system (Luminex, Austin, TX, USA).

### Microscopy and Analysis

Retinal cultures were imaged using an LSM700 laser scanning confocal microscope (Carl Zeiss, Inc., Thornwood, NY, USA). For rat retinal cultures, the entire eight-well chamber was images and the number of βIII-tubulin^+^ RGC/RGC with neurites was quantified. For hRGC cultures, 1-mm^2^ scans were collected and the number of BRN3B^+^ RGCs was quantified. Counts were conducted manually by an individual masked to the treatment groups. All cell cultures (rat and human) were performed both in triplicate and three separate times (*n* = 3).

### Statistics

Animal numbers were determined beforehand using a power calculation (Gpower). All statistical tests were performed using SPSS 17.0 (IBM SPSS, Inc., Chicago, IL, USA) and data presented as mean ± standard error of the mean (SEM) with graphs constructed using SigmaPlot (San Jose, CA, USA). The Shapiro-Wilkes test was used to ensure all data were normally distributed before parametric testing using a one-way analysis of variance (ANOVA) with a Tukey post hoc test. Statistical differences were considered significant at *P* values <0.05. For the quantitative real-time polymerase chain reaction (qRT)-PCR, data were compared by a Student's *t-*test and statistical significance set at *P* < 0.001.

## Results

### MSC Conditioned Medium Is Neuroprotective in Adult Rat Retinal Cultures, but Only When Primed

**Figure 2.**
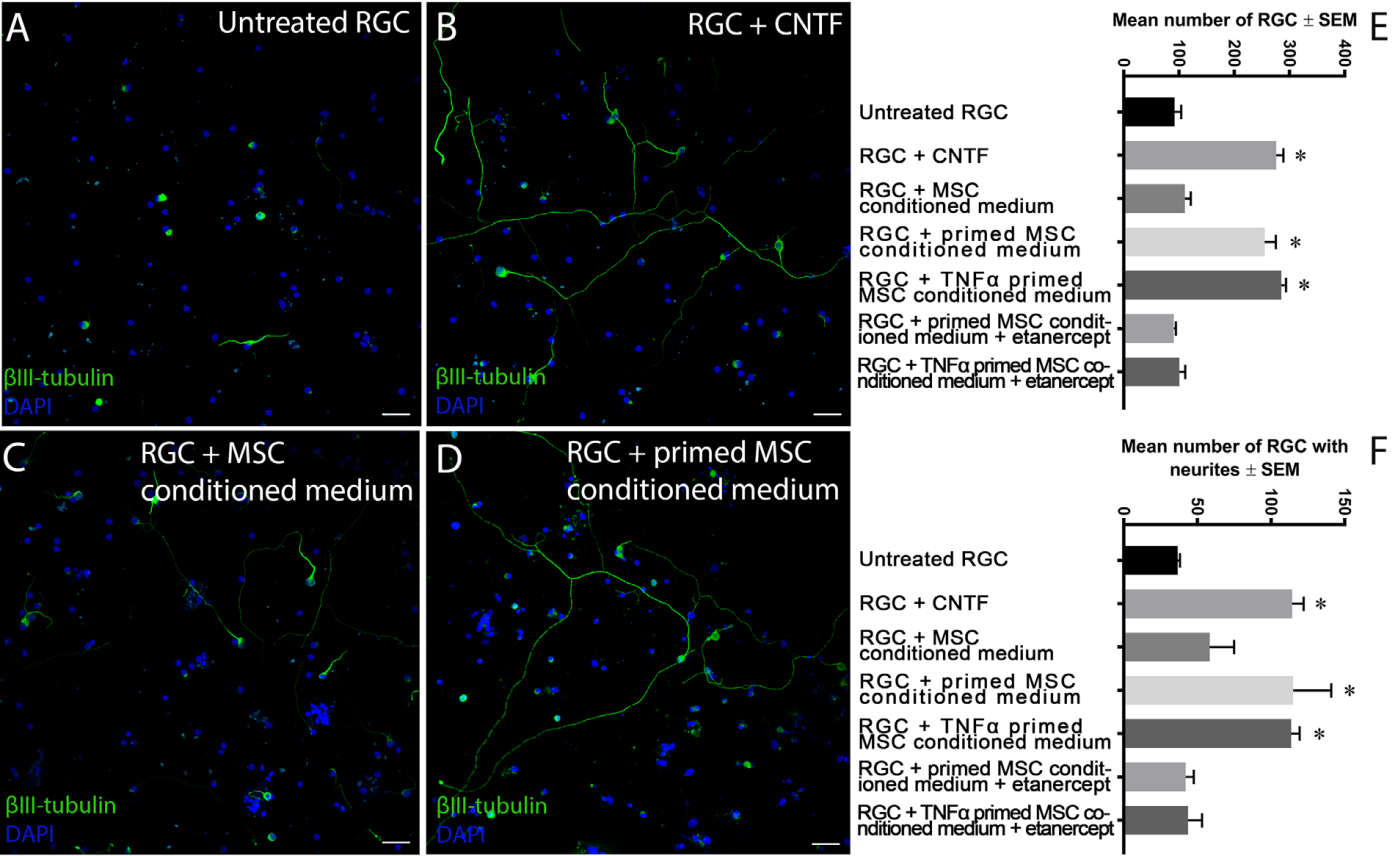
MSC conditioned medium treatment of heterogenous retinal cultures. Heterogenous adult rat retinal cultures were left for three days either untreated (negative control; **A**), treated with CNTF (positive control; **B**), treated with MSC conditioned medium (**C**), treated with primed MSC conditioned medium without (**D**) or with etanercept (TNFα blocker) added during priming, or treated with TNFα primed MSC conditioned medium without or with etanercept added during priming (*scale bar*: 50 µm). The number of RGCs (**E**), denoted by their asymmetrical βIII-tubulin staining, and the number of RGCs with neurites (**F**) were quantified (*n* = 3). *Asterisks* indicate a significant increase in RGC neuroprotection/neuritogenesis in comparison to untreated control/unprimed MSC conditioned medium (*P* < 0.05) and includes CNTF treatment and primed MSC (via retinal medium or TNFα) conditioned medium treatment.

We first determined the neuroprotective and neuritogenic properties of retinal cell culture and TNFα-primed MSC conditioned medium in freshly isolated rat retinal cells. In untreated controls, 92 ± 12 (mean ± SEM) RGCs survived with 36.7 ± 1.5 RGCs containing neurites (Figs. 2A, 2E, 2F). Retinal cells cultured with the positive control CNTF showed 276.3 ± 12.7 surviving RGCs and 113.7 ± 5.7 RGCs with neurites (Figs. 2B, 2E, 2F) while treatment of retinal cells with unprimed MSC conditioned medium promoted the survival of 110.7 ± 10.7 RGCs and 58.3 ± 16.6 RGCs with neurites (Figs. 2C, 2E, 2F). However, treatment of retinal cells with retinal cell culture–primed MSC conditioned medium (referred to simply as “primed MSC conditioned medium”) elicited significant neuroprotection (255 ± 20.5 RGCs) and neuritogenesis (115 ± 25.9 RGCs with neurites) (Figs. 2D, 2E, 2F) compared with unprimed MSC conditioned medium and untreated controls (Fig. 2). Significant neuroprotection and neuritogenesis, similar to primed MSC conditioned medium, was also achieved after treatment with conditioned medium from MSCs primed with TNFα (285.3 ± 8.5 RGCs and 113.7 ± 5.7 RGCs with neurites, respectively) (Figs. 2E, 2F), and both priming methods were equivalently neuroprotective and neuritogenic as CNTF-treated positive control cultures. If etanercept, a potent blocker of TNFα, was included in the MSC culture during priming, the primed MSC conditioned medium (primed via treatment with rat retinal cultures or TNFα) no longer elicited neuroprotection (90.7 ± 3.8 and 100.7 ± 10.8 RGCs, respectively) or neuritogenesis (42 ± 5.6 and 43.7 ± 9.5 RGCs with neurites, respectively) (Figs. 2E, 2F). These results demonstrate that both primed MSC conditioned medium and MSC conditioned medium primed with TNFα cause significant RGC protection and neurite outgrowth. In addition, the ablation of priming by etanercept demonstrates that retinal conditioned medium primed MSCs primarily through TNFα.

### MSC Exosomes Are Neuroprotective in hRGC Cultures and More Effective After Priming

**Figure 3.**
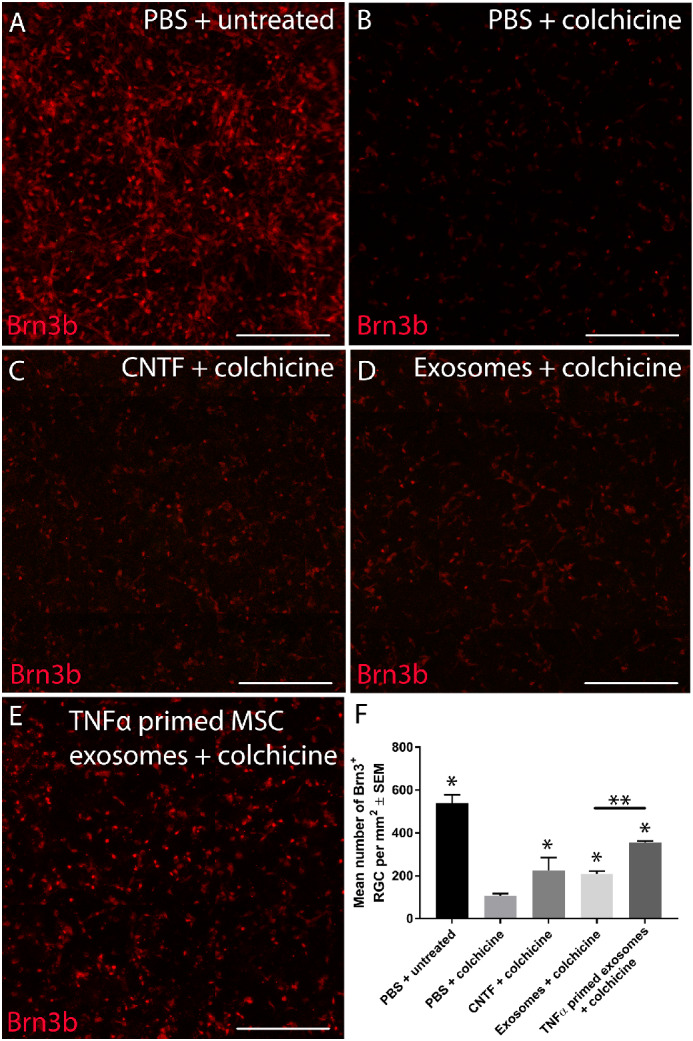
Exosome treatment of heterogeneous human stem cell–derived retinal cultures. Heterogeneous retinal cultures derived from human stem cells were left uninjured/untreated (**A**), injured with colchicine but untreated (negative control; **B**), injured with colchicine/treated with CNTF (positive control; **C**), injured with colchicine/treated with MSC exosomes (**D**), or injured with colchicine/treated with TNFα primed MSC exosomes (**E**; *scale bar*: 250 µm). The number of RGCs (**F**), identified by the Brn3b expression, were quantified (*n* = 3). *Asterisks* indicate significant difference from untreated/colchicine-injured control at *P* < 0.05). *Asterisks* also indicate a significant increase in RGC neuroprotection in comparison to untreated/colchicine-injured control (*P* < 0.05) and include CNTF treatment, exosome treatment, and TNFα primed exosome treatment. Furthermore, significant difference was also observed between exosome treatment and TNFα primed exosome treatment (*double asterisks*).

We next determined if MSC exosomes were neuroprotective to hRGCs and whether priming with TNFα potentiated the neuroprotective effects. Whereas rodent retinal cultures degenerate spontaneously in culture, hRGCs require colchicine to trigger their degeneration.^31^^,^^32^ In untreated cells + vehicle (PBS), abundant BRN3B^+^ hRGCs were detected in culture (Figs. 3A, 3F) while the addition of colchicine to hRGC cultures caused an approximate 80% loss of hRGCs (107.5 ± 9.3 hRGCs; Figs. 3B, 3F) compared with uninjured cultures (539 ± 39 hRGCs; Figs. 3A, 3F). Treatment with MSC exosomes led to significant hRGC neuroprotection (208.1 ± 14.2 hRGCs; Figs. 3D, 3F) compared with untreated, colchicine-injured cultures and was equivalent to CNTF-treated positive control cultures (224.8 ± 60.7 hRGCs; Figs. 3C, 3F). Moreover, treatment with TNFα-primed MSC exosomes led to a significantly enhanced neuroprotective effect (355.7 ± 7.3 hRGCs) compared with MSC exosome treatment (Figs. 3E, 3F). These results demonstrate that MSC exosomes and TNFα-primed MSC exosomes provide significant neuroprotection to hRGCs cultured in the presence of “injury-inducing” colchicine.

### Retinal Conditioned Medium, Used to Prime MSCs, Contains TNFα

**Figure 4.**
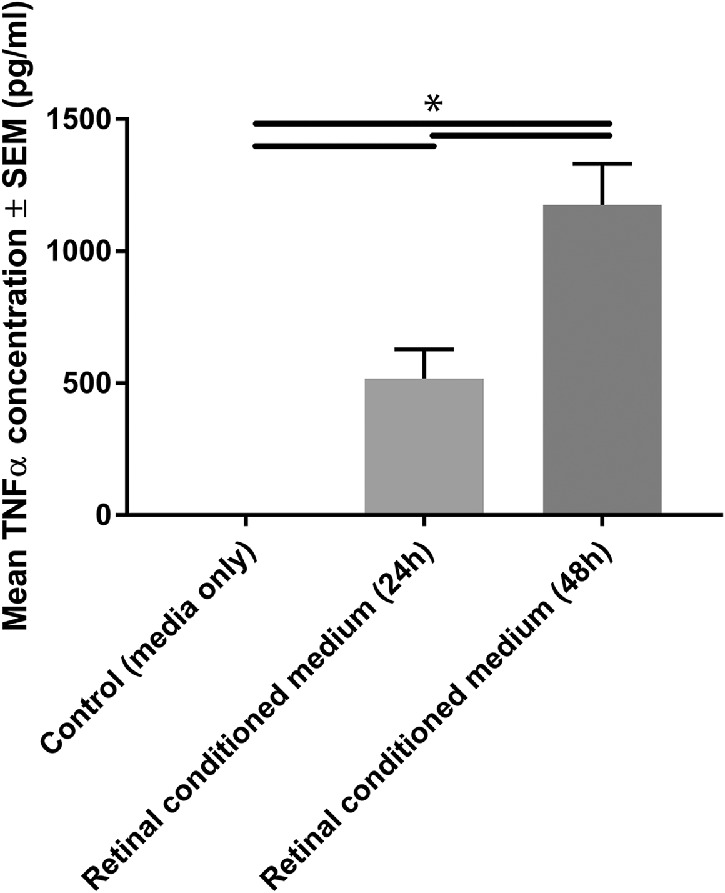
TNFα secretion by heterogeneous retinal culture. Retinal culture conditioned medium, collected after 24 or 48 hours in culture (or unconditioned controls), was assayed using a TNFα ELISA (*n* = 3; *black lines* indicate significant difference at *P* < 0.05).

With retinal conditioned medium reliably priming MSC and etanercept, a TNFα blocker, inhibiting priming, we next sought to confirm that conditioned medium from retinal cell cultures contained TNFα. TNFα was absent from supplemented Neurobasal-A, but significant concentrations were observed after retinal cell culture conditioning for 24 hours (518 ± 109.9 pg/mL) and 48 hours (1175.4 ± 154.9 pg/mL; Fig. 4). These results demonstrate that significant titers of TNFα were present in conditioned medium from retinal cell cultures.

### Priming Had No Effect on Division/Exosome Secretion Rate

**Figure 5.**
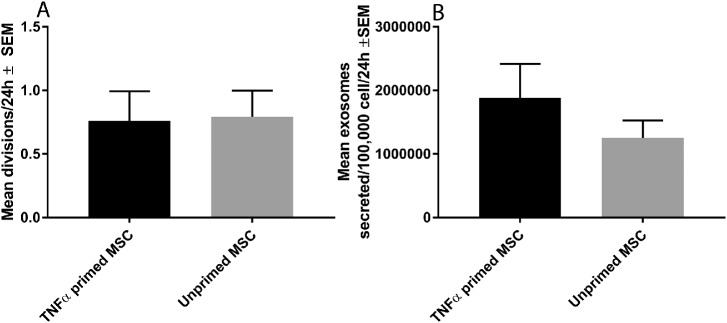
Division and exosome secretion rate of primed MSCs. (**A**) The mean number of divisions of unprimed MSCs and those primed by TNFα. (**B**) The mean number of exosomes secreted by unprimed MSCs and those primed by TNFα (*n* = 3, no significant differences observed at *P* > 0.05).

We then investigated if priming MSCs with TNFα altered their division rate or secretion of exosomes. MSC division rate following TNFα priming (0.76 ± 0.23 divisions/24 hours) was not significantly different from the division rate of unprimed MSCs (0.79 ± 0.21 divisions/24 hours; Fig. 5A). MSC exosome secretion rate following TNFα priming (1.88 * 10^6^ ± 5.34 * 10^5^ exosomes/100,000 cells/24 hours) trended toward but was not significantly higher than the exosome secretion rate of unprimed MSCs (1.25 * 10^6^ ± 2.75 * 10^5^ exosomes/100,000 cells/24 hours; Fig. 5B). These results showed that MSC division rate and secretion of exosomes were unaffected by priming.

### Priming MSCs Significantly Increases Exosomal PEDF, VEGF-A, and Platelet-derived growth factor (PDGF)-AA

**Figure 6.**
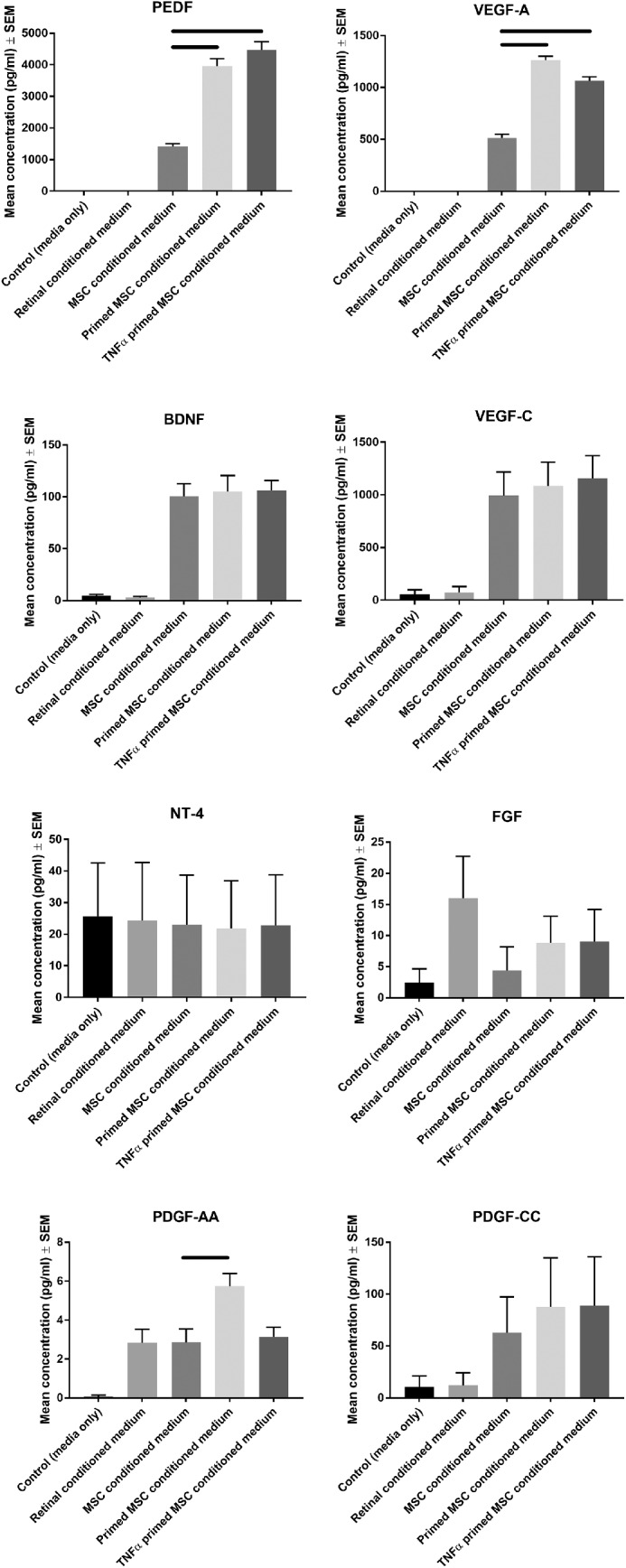
Luminex analysis of primed MSC protein secretome. Luminex analysis was performed on control MSC growth media, control retinal conditioned medium, unprimed MSC conditioned medium, primed MSC conditioned medium, and TNFα primed conditioned medium (*n* = 3; *black lines* indicate significant differences between primed and unprimed at *P* < 0.05). Beta-NGF, PDGF-DD, PDGF-BB, EGF, VEGF-D, and PDGF-AB were not detected.

We next determined the abundance of a number of NTFs in exosome preparations from MSC conditioned medium, retinal cell culture conditioned medium, and primed MSC conditioned medium. We observed that PEDF abundance was significantly enhanced in primed MSC conditioned medium and TNFα primed conditioned medium (3693 ± 232 pg/mL and 4469 ± 261 pg/mL, respectively) compared with unprimed MSCs (1408 ± 93 pg/mL; Fig. 6). VEGF-A abundance was also significantly enhanced in MSC conditioned medium and TNFα primed conditioned medium (1263 ± 38 pg/mL and 1063 ± 40 pg/mL, respectively) compared with unprimed MSCs (514 ± 37 pg/mL). PDGF-AA abundance was significantly enhanced after priming (5.74 ± 0.65 pg/mL) but not after TNFα priming (3.15 ± 0.49 pg/mL) compared with unprimed MSCs (2.86 ± 0.69 pg/mL). Brain-derived neurotrophic factor (BDNF), VEGF-C, NT-4, FGF, and PDGF-CC were detectable but not significantly increased after priming/TNFα priming. Beta-NGF, PDGF-DD, PDGF-BB, Epidermal growth factor (EGF), VEGF-D, and PDGF-AB were possible analytes in the present assay but were not detected.

## Discussion

The present study demonstrates that MSCs are receptive to priming following their introduction to an injured environment. In the case of the injured retina, MSCs are primed due to the release of TNFα. After priming, MSCs exert a significantly greater neuroprotective effect on injured RGCs, both rat and human derived. Congruent with our previous work, this effect is paracrine mediated,^8^ likely through their released exosomes.^33^ The augmented effect TNFα priming afforded MSC exosomes possibly involves an increase in exosomal NTFs.

TNFα acts through two receptors, TNF receptors 1 and 2, with receptor 2 being the likely candidate for its actions on neural cells.^34^ Previous work has demonstrated that stimulation of MSCs by TNFα significantly upregulated production of NTFs, including VEGF, Hepatocyte growth factor (HGF), and Insulin-like growth factor (IGF).^35^ Mitogen-activated protein kinase (MAPK) inhibitors decreased the production of these NTFs while they had no effect on baseline secretion, suggesting the upregulated secretion is a distinct mechanism. In a separate study, MSCs exposed to either hypoxic conditions or TNFα are activated through the NF-κB pathway and upregulate their secretion of several NTFs, including VEGF, FGF2, IGF-1, and HGF.^36^ As well as secretion of NTFs, migration into injured tissue is an important property integral to the regenerative potential of MSCs. Treatment of MSCs with TNFα increased their invasion and migration through the extracellular matrix, attributed partially to the upregulation of intracellular adhesion molecule 1^37^ and vascular cell adhesion protein 1.^38^ In the present study, we demonstrated that retinal conditioned medium was ineffective at priming MSCs if etanercept, a TNFα inhibitor, was included, suggesting TNFα is a critical mediator in priming MSCs in this model.

Both PEDF and VEGF-AA, which were found to be significantly upregulated after TNFα priming of MSCs, are known neuroprotective agents for injured RGCs. Müller cells exert a neuroprotective effect on RGCs through the secretion of PEDF,^39^ while delivery of PEDF into retinal cultures or rat eyes after optic nerve crush leads to significant RGC neuroprotection.^40^ Delivery of VEGF into ocular hypertensive eyes leads to significant RGC neuroprotection while also upregulating PEDF.^41^ A separate study in an ocular hypertensive model demonstrated that VEGF-A promoted RGC neuroprotection via direct activation of the VEGF receptor 2 and signaling through the phosphoinositide-3-kinase/Akt pathway.^42^ The same group showed that treating the same primary retinal cultures used in this study with VEGF-A elicited 50% more RGC neuroprotection than untreated controls. While comparative analysis is difficult as the referenced study used hypoxia to injure the cultured RGCs, exosomes appear to be more efficacious, likely due to their cargo containing multiple NTFs. In comparison to VEGF, PEDF appears to act, at least partially, through retinal glia, leading to suppression of RGC caspase 2.^43^ Treatment of RGC cultures with PEDF leads to a 63% increase in RGC survival in comparison to untreated cultures, which, similar to VEGF-A, cannot account for the full neuroprotective effect elicited by exosomes. PDGF-AA is also a potent neuroprotective for RGCs and an integral component of the neuroprotective secretome of MSCs.^9^ Interestingly, while PDGF-AA abundance in or associated with exosomes was upregulated following MSC priming with retinal conditioned medium, this was not seen when MSCs were primed with TNFα. While the present study focused on TNFα, it is apparent that other injury-associated cytokines are involved in the priming of MSCs. While these NTFs were identified in our exosome samples, it is possible they were not in the cargo but rather externally associated with the exosomes. A previous study performing proteomic analysis of extracellular vesicles demonstrated the presence of PEDF, with the authors suggesting that the protein is extracellular and associated with the exosome.^44^ Further study is required to confirm the importance of these cytokines in the improved neuroprotective efficacy of primed MSCs.

We tested our samples in two in vitro models of RGC death. The first was a primary culture of adult rat RGCs. We have already demonstrated the neuroprotective efficacy of MSC exosomes in this model,^33^ and thus, it served as a reliable system with which to compare the improved efficacy of primed MSC exosomes/conditioned medium. The second in vitro model was hRGCs derived from human stem cell cultures.^31^^,^^32^ To our knowledge, this is the first time exosomes have been used as a therapeutic on hRGCs, and our results not only reinforce our previous findings in rodent models but also add credibility to the improved therapeutic efficacy after priming.

In conclusion, the present study demonstrated the feasibility and ease by which the efficacy of MSCs and their exosomes can be improved. TNFα is one such priming candidate, and the effects appear to involve the upregulation of neurotrophic proteins that are released in, or associated with, exosomes. Finally, we build upon the established literature demonstrating a neuroprotective efficacy of MSC exosomes by showing that their potential also extends to human retina. Future studies will be directed toward improving exosomes’ efficacy while ensuring their safety and tolerance, having translation into patients as the rapidly approaching goal.
